# Hybrid Block-Based Lightweight Machine Learning-Based Predictive Models for Quality Preserving in the Internet of Things- (IoT-) Based Medical Images with Diagnostic Applications

**DOI:** 10.1155/2022/8173372

**Published:** 2022-04-12

**Authors:** V. K. Reshma, Ihtiram Raza Khan, M. Niranjanamurthy, Puneet Kumar Aggarwal, S. Hemalatha, Khalid K. Almuzaini, Enoch Tetteh Amoatey

**Affiliations:** ^1^Department of Artificial Intelligence and Machine Learning, Hindusthan College of Engineering and Technology, Malumichampatti, India; ^2^Department of Computer Science, Jamia Hamdard, New Delhi, India; ^3^Department of Computer Applications, M S Ramaiah Institute of Technology (Affiliated to Visvesvaraya Technological University, Karnataka), Bangalore, India; ^4^Department of Information Technology, ABES Engineering College, Ghaziabad, Uttar Pradesh, India; ^5^Department of Computer Science and Engineering, Panimalar Institute of Technology, Chennai, Tamil Nadu, India; ^6^National Center for Cybersecurity Technologies (C4C), King Abdulaziz City for Science and Technology (KACST), Riyadh 11442, Saudi Arabia; ^7^School of Engineering, University for Development Studies, Tamale, Ghana

## Abstract

In the contemporary era of unprecedented innovations such as the Internet of Things (IoT), modern applications cannot be imagined without the presence of a wireless sensor network (WSN). Nodes in WSN use neighbor discovery (ND) protocols to have necessary communication among the nodes. The neighbor discovery process is crucial as it is to be done with energy efficiency and minimize discovery latency and maximum percentage of neighbors discovered. The current ND approaches that are indirect in nature are categorized into methods of removal of active slots from wake-up schedules and intelligent addition of new slots. This work develops a lightweight intrusion detection system (IDS) based on two machine learning approaches, namely, feature selection and feature classification, in order to improve the security of the Internet of Things (IoT) while transferring medical data through a cloud platform. In order to take advantage of the comparatively cheap processing cost of the filter-based technique, the feature selection was carried out. The two methods are found to have certain drawbacks. The first category disturbs the original integrity of wake-up schedules leading to reduced chances of discovering new nodes in WSN as neighbors. When the second category is followed, it may have inefficient slots in the wake-up schedules leading to performance degradation. Therefore, the motivation behind the work in this paper is that by combining the two categories, it is possible to reap the benefits of both and get rid of the limitations of both. Making a hybrid is achieved by introducing virtual nodes that help maximize performance by ensuring the original integrity of wake-up schedules and adding efficient active slots. Thus, a Hybrid Approach to Neighbor Discovery (HAND) protocol is realized in WSN. The simulation study revealed that HAND outperforms the existing indirect ND models.

## 1. Introduction

This is an emerging paradigm in the world of computer networks, which permits communication between all sorts of items over the Internet [1]. It is referred to as the Internet of Things (IoT). These objects, which may include RFID tags, sensors, actuators, mobile phones, and other similar devices, all communicate and collaborate using a single addressing method in order to accomplish a shared purpose. The Internet of Things (IoT) enables the combination of all types of communications, all of the time, for everyone, and on any device, resulting in ubiquitous computing [2]. It will cover a broad variety of applications and will touch on practically all of the issues we deal with on a daily basis, according to the authors.

IoT devices are often deployed in a hostile and unsecured environment, making them more susceptible to a variety of threats. As a result, security solutions are required to safeguard Internet of Things devices against intruder assaults [3]. When an intrusion detection system (IDS) detects an attack on a system or a network, it analyzes the activities and events that occurred during the assault. It has the potential to serve as a second line of protection against the invader. The primary goal of an intrusion detection system (IDS) is to identify as many assaults as feasible with acceptable accuracy while using the least amount of energy possible in resource-constrained environments [4]. There are two kinds of intrusion detection systems: signature-based and anomaly-based. Intruders are detected via a signature-based intrusion detection system, also known as a misuse-based IDS, which compares fresh data with a knowledge base or signatures of previously detected assaults [5]. This method is effective at detecting known assaults, but it is ineffective at detecting novel attacks. The anomaly-based intrusion detection system compares actions that are deemed typical with observed occurrences in order to discover substantial deviations [6].

Many studies have been conducted lately in the fields of IoT and intrusion detection systems (IDSs) in order to give the finest security mechanism. A light anomaly detection approach based on the notion of game theory piqued their attention. The Nash equilibrium is used by the authors to anticipate the equilibrium state that will enable the IDS agent to detect the signature of a new attack signature. The article [7] introduced a novel intrusion detection system in a wireless sensor network that is based on the k-Nearest Neighbor (KNN) classification technique and uses the KNN classification algorithm. In the wireless sensor network, the system is capable of detecting a flood assault. As part of its research, it also performs trials to determine the impacts of a flood. Reference [8] introduced a distributed internal anomaly detection system for the Internet of Things, which is a distributed internal anomaly detection system. The monitoring, rating, isolation, and reporting functions are the most important components of the system. As part of their one-hop monitoring, nodes keep track of their neighbors' ratings, and if a neighbor fails to keep up with the needed rating, the surrounding node is labeled as an anomaly. SVELTE is a real-time intrusion detection solution for the Internet of Things suggested [9]. It is an IDS that is accessible in the Internet of Things and has been incorporated in the Contiki OS. Using this method, only content spoofing attacks inside the network, as well as gulp and selective transfer assaults, are detected. Several researchers, including [9, 10], have described an ultralightweight deep-packet anomaly detection technique that may be implemented on tiny Internet of Things sensors. The technique models payloads using n-gram bit-patterns and enables the size of the n-grams to change depending on the dimension.

However, even though all of the aforementioned research asserts that a detection system has been implemented and that some attacks have been successfully detected, it is necessary to make the detection system lightweight if we are to implement an efficient detection system in the Internet of Things environments.

The goal of our study is to develop a low-weight IDS system. Two machine learning approaches, feature selection and classification methods, have been used to achieve this goal: feature selection and classification methods. Feature selection techniques may be used to pick relevant characteristics that can be utilized to reduce computational and storage costs while also improving the overall accuracy of detection. When it comes to feature selection, there are three primary ways [10]: filter-based, wrapper-based, and embedded-based approaches. The filter approach was employed in this work because it has a low computational cost when compared to the wrapper and embedded methods. Additional to this, the Scikit-Learn tool has been used to build numerous common algorithms like Decision Trees, k-Nearest Neighbor, Support Vector Machines, and others, in order to discover the best classification model fit for the IoT context [11].

A comparative comparison of feature selection approaches and their impacts on various classification algorithms is shown at the conclusion of this paper, which uses three distinct data sets, including the KDD99, the NSL-KDD, and the UNSW-NB15 datasets [12].

In the contemporary era of unprecedented innovations such as the Internet of Things (IoT), modern applications cannot be imagined without the presence of a wireless sensor network (WSN) [13]. Nodes in WSN use neighbor discovery (ND) protocols to have necessary communication among the nodes [14]. The neighbor discovery process is crucial as it is to be done with energy efficiency [15] and minimize discovery latency and maximize the percentage of neighbors discovered [16]. There are many existing ND protocols for continuous ND. Kumar et al. [1] studied the WSNs associated with IoT use cases for ascertaining ND challenges. Elhabyanet et al. [2] focused on the ND coverage protocols associated with WSN. Chen and Bai [17] explored EQS systems on ND and rendezvous maintenance. Lee et al. [18] proposed a prime set-based approach toward ND for low-duty-cycled WSN. Bansal et al. [19] built an efficient ND protocol for low-duty-cycled WSN. Bakht et al. [20] proposed a wake scheduling mechanism based on heterogeneous quorum. Another quorum-based approach is proposed by Saraereh et al. [21] with a weighted approach besides rendezvous consistency. Wei et al. [14] discussed the working of ND protocol known as Searchlight. Chen and Bai [17] explored EQS systems on ND and rendezvous maintenance. Lee et al. [18] proposed a prime set-based approach toward ND for low-duty-cycled WSN. Wei et al. [14] built an efficient ND protocol for low-duty-cycled WSN. Wei et al. [22] proposed a wake scheduling mechanism based on heterogeneous quorum. Another quorum-based approach is proposed by Saraereh et al. [21] with a weighted approach besides rendezvous consistency.

From the literature, it is understood that there are many ND protocols [23]. With respect to indirect ND protocols, there are two important groups. One group of protocols focused on adding new active slots in wake-up schedules while the other category removed certain active slots. The first category disturbs the original integrity of wake-up schedules leading to reduced chances of discovering new nodes in WSN as neighbors. When the second category is followed, it may have inefficient slots in the wake-up schedules leading to performance degradation [17]. Therefore, the motivation behind the work in this paper is that by combining the two categories, it is possible to reap the benefits of both and get rid of the limitations of both. Our contributions in this paper are as follows.

1. We proposed a Hybrid Approach to Neighbor Discovery (HAND) protocol which combines two indirect ND methods [18] to reap their benefits besides overcoming their existing limitations.
2. We proposed an algorithm to realize the notion of a virtual node and its mechanisms to ensure performance improvement in the HAND protocol.
3. We have built a simulation prototype to evaluate the HAND protocol and its underlying mechanisms [19] in ND.
4. We use Hybrid Method to improve the expected output of semi-structured sequential data. We train and test the work on data that is outside either an Artificial Neural Network or a state machine's normal capability with simplified notation extracted from midi files [20]. A Hybrid Model is constructed by implementing multiple applicable machine learning algorithms such as the SVM model and Bayesian's Classification model or any other models in order to overcome drawbacks faced by each other and also provide their mutually contributed efficiency [22].

The remainder of the paper is structured as follows.  Section 2 reviews literature on existing ND methods.  Section 3 presents the HAND protocol.  Section 4 presents experimental results.  Section 5 concludes the paper and gives information about the future scope of the research.

## 2. Related Work

Different ND protocols and related works are found in the literature. Kumar et al. [1] studied the WSNs associated with IoT use cases for ascertaining ND challenges. Elhabyanet al. [2] focused on the ND coverage protocols associated with WSN. Shukla et al. [3] investigated the methods used for energy efficiency in the field environment. Deng et al. [4] studied services in WSN for improving concurrent compositions. Zhou et al. [5] focused on WSN usage in real-world applications such as pollution monitoring. Onyema et al. [6], on the other hand, explored data collection methods for WSN. Yi et al. [7] and Xie et al. [8] focused on vegetable greenhouses and industrial monitoring with enhanced performance using WSNs. Shukla et al. [9] proposed and discussed many fast ND algorithms. Almeida et al. [16] explored fractal clustering and Pearson correlation for enhancing the lifetime of WSN. Aponte-Luis et al. [11] investigated mobile sensing applications to observe ND mechanisms. Tomar and Shukla [12] focused on asynchronous wake schedules in WSN using Prime Block Design (PBD). Wei et al. [14] discussed the working of ND protocol known as Searchlight.

Almeida et al. [16] explored an ND protocol for IoT using a probabilistic neighborhood model for leveraging performance. Zhang et al. [24] focused on generic approaches that could improve ND performance in mobile sensor applications. Zhang et al. [25] explored EQS systems on ND and rendezvous maintenance. Lee et al. [18] proposed a prime set-based approach toward ND for low-duty-cycled [26] WSN. Bansal et al. [19] built an efficient ND protocol for low-duty-cycled WSN. Bakht et al. [20] proposed a wake scheduling mechanism based on heterogeneous quorum. Another quorum-based approach is proposed by Wei et al. [22] with a weighted approach besides rendezvous consistency. Kandhalu et al. [27] proposed U-connect, an ND protocol while Meng et al. Reference [28] proposed code-based ND protocol. Zhang et al. [28] focused on asynchronous neighbor discovery for mobile sensor devices. A similar kind of work was carried out by Shukla et al. [29] while Kindt et al. [30] proposed yet another protocol named Griassdi for ND. Wei et al. [31] proposed an energy-efficient ND while a similar kind of work is carried out by Suarez and Nayak [32]. Cai and Wolf [33] proposed an ND protocol based on quorum and self-adaptive in nature. Device discovery in mobile computing environments is studied in [34, 35].

Other important approaches found in the literature include ND for mobile WSN [27, 36], RSSI for distance measure [28], self-adaptive ND [37], proactive ND for mobiles [38], ND with slot length control [39], ND with multipacket reception model [30], ND model known as Panacea [40], prime number-based ND [41], and group-based ND [32]. Secure critical data reclamation methods for isolated clusters in WSN were proposed in [33, 42]. From the literature, it is understood that there are many ND protocols. With respect to indirect ND protocols, there are two important groups. One group of protocols focused on adding new active slots in wake-up schedules while the other category removed certain active slots [43]. The first category disturbs the original integrity of wake-up schedules leading to reduced chances of discovering new nodes in WSN as neighbors. When the second category is followed, it may have inefficient slots in the wake-up schedules leading to performance degradation [44]. Therefore, the motivation behind the work in this paper is that by combining the two categories, it is possible to reap the benefits of both and get rid of the limitations of both.

## 3. Proposed Hybrid Approach to Neighbor Discovery

The use of Internet of Things (IoT) technology in healthcare applications assures that the healthcare sectors may improve the quality of care while also reducing costs thanks to the automation and resource optimization offered by the technology [45]. Because of the Internet of Things (IoT) in medical imaging, it is possible to identify problems and take remedial steps in real time, as well as to perform automatic analysis of the imaging equipment parameters with ease. When it comes to medical technology [21], digitization has reached many sectors, including monitoring and control of medical equipment. The Internet of Things in medical imaging will minimize the waiting time and annoyance for both the patients and the doctors. As a result, the article includes a review of Internet of Things-based medical imaging technologies for healthcare applications [46], as well as an explanation of the relevance of the Internet of Things in the field of medical imaging.

**Figure 1:**
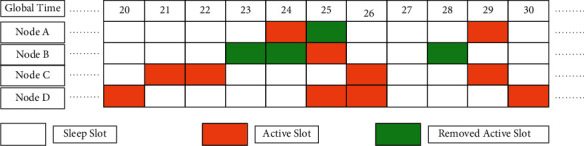
Modus operandi of NAND with Disco as a pairwise method.

The current ND approaches that are indirect in nature are categorized into methods of removal of active slots from wake-up schedules and intelligent addition of new slots [47]. The two methods are found to have certain drawbacks. The first category disturbs the original integrity of wake-up schedules leading to reduced chances of discovering new nodes in WSN as neighbors. When the second category is followed, it may have inefficient slots in the wake-up schedules leading to performance degradation [48]. Therefore, the motivation behind the work in this paper is that by combining the two categories, it is possible to reap the benefits of both and get rid of the limitations of both. Making a hybrid is achieved by introducing virtual nodes that help maximize performance by ensuring the original integrity of wake-up schedules and adding efficient active slots [49]. The proposed protocol named Hybrid Approach to Neighbor Discovery (HAND) is motivated by the facts aforementioned. It overcomes the limitations of existing methods like the Extended Quorum System (EQS) which is energy-efficient in discovering neighbors. However, in the process of removing active slots, it incorrectly removes them and thus it cannot discover new neighbors efficiently. By using a hybrid approach that considers both intelligent [50] addition and removal of active slots in the wake-up schedules, HAND protocol gains benefits of the two indirect approaches that focus on adding new slots and removal existing slots, respectively. The modus operandi of the HAND protocol is illustrated in Figure 1.

In essence, HAND tries to remove active slots that lower energy efficiency [51] and ensures the discovery of new neighbors. The active slots are arranged in global time from 20 through 30 for different nodes. Based on a distance threshold of 20 m, nodes that exhibit a smaller distance between them can have more common neighbors. Thus, they form a group as discussed in Section 3. Such groups are cooperative groups. Assume that the distance between nodes *A* and *B* is 15 m, that between *B* and *C* is 25 m, and that between *A* and *C* is 30 m. As per the threshold, both *A* and *B* are a group where nodes *A* and *B* share a task of activating a node using global slots. The HAND follows a hybrid of indirect methods besides using a paired method [52]. In a given cooperative group, the duty cycles are the same. Any node's wake-up plan in the given group is the plan of such group. When randomly selected, the active slots for node *A* are 24 and 29 while slot 25 is the active slot for node *B*. In slot 25, node *A* remains in sleep mode while node *B* is in sleep mode in slots 23, 24, and 28. A removed active slot in the original wake-up schedule is considered removed and now it becomes a sleep slot. Node *C* works as per the Disco method. After slot 20, when *D* is the neighbor of *A*, *B*, and *C*, in slot 25, both *B* and *D* discover each other. In slot 26, bot *C* and *D* discover each other. *B* broadcasts messages saying that it is very close to *A*. As per the spatial similarity of neighbors, concept *D* considers *A* as its neighbor. Thus, in summary, ND is improved significantly. In the case of EQS, the drawback is that *D* cannot discover other nodes. Therefore, HAND improves the neighbor discovery percentage. In the HAND protocol, the distance between nodes is obtained using RSSI as expressed in

(1) $$\begin{array}{c} d_{AB}=10^{RSSI_{AB}-RSSI_{\max}/10m}, \end{array}$$

where *m* denotes path loss exponent while RSSImax denotes the maximum RSSI. The HAND protocol works together with pairwise methods like Disco and Searchlight. Based on the distance threshold, two nodes can form a cooperative [53] group. Group formation and group cooperation are the two important phases in the proposed protocol. They are computed as in equations (2) and (3), respectively.

(2) $$\begin{array}{c} T_{1}=\left(\left|\log_{2}\left(n_{opt}\right)\right|-1\right)t\varepsilon +\frac{nopt^{-2\left|\log_{2}n_{opt}\right|}}{2^{\left|\log_{2}n_{opt}\right|-1}}t\varepsilon , \end{array}$$

(3) $$\begin{array}{c} T_{2}=\frac{d_{TV2}-C_{0}-C_{1}n-C_{2}d_{0}}{C_{3}+C_{4}n}. \end{array}$$

The *T*_1_ and *T*_2_ steps are used in the proposed algorithm below. They are used to determine the respective times such as group formation time and group cooperation time.

The proposed algorithm is meant for achieving better performance with HAND protocol with respect to neighbor discovery.

As presented in Algorithm 1, group formation and group cooperation are controlled in order to have a discovery of neighbors based on the HAND protocol. It has a number of iterative processes that ensure efficient neighbor discovery [54]. The nodes in the WSN are in a movement model throughout the process. Thus, the groups' formation is dynamic in nature. Each group where the members are in the 20 m range is known as a virtual node. This is an essential phenomenon in the HAND protocol. Based on this threshold, the algorithm takes care of the making and management of groups. When nodes move away from a group, or when a group is dissolved, the nodes can have their individual wake-up schedules and still wait for an opportunity to form a group for continuous neighbor discovery [55].

## 4. Experimental Results

### 4.1. Data Collection

As data collecting sources for device-level detection, we take into consideration both wireless sensing devices (WBAN) and medical imaging equipment. Data is acquired from each of these sources, but in a separate and independent manner [56]. The data collecting procedure makes use of benign network circumstances in order to avoid the model from being contaminated by malicious data during the gathering phase. For sensor devices, the information they collect is retrieved as a tuple including a timestamp and a sensor scalar value, while the information they collect from image devices [57, 58] is changed in real time from a pixel matrix to an integer scalar value.

In this part, we will detail our simulation setup, attack models, and evaluation metrics, among other things. We emulate the Internet of Medical Things, which is made up of heterogeneous devices that communicate with one another via a variety of network protocols [59]. We explore a combination of wireless sensing devices that are either Zigbee-enabled or that are compliant with the 802.15.6 WBAN protocol. Our system also incorporates additional intelligent and linked devices, such as ultrasound scanners and magnetic resonance imaging (MRI) equipment that operate on the DICOM network protocol. In order to handle the variance in communication protocol standards, we make certain that the suggested system is built to be completely ignorant to the variations between network standards [60]. Our simulations were carried out using the OMNeT-based Castalia-3.2 simulator, which was specifically designed for wireless body area networks. Besides emulating network implementations throughout the whole protocol stack with complete customizability, the simulator also includes preloaded IEEE 802.15.6 and 802.15.4 implementations for testing. Its built-in radio model is also capable of simulating both constructive and destructive interference, RX to TX to sleep transitions, and energy use calculations, among other things.

Simulations were out with the help of a WSN network placed in a 500 × 500 area. The network is split into a grid with sides that are each 10 meters in length. Nodes near the boundaries of the grid are deemed to have mobility, according to this definition. Since the suggested protocol requires a slot length of 25 ms, this duration has been considered for simulation [60]. It has the ability to prevent collisions while also eliminating clock jitters. HAND's experimental findings are compared to those obtained by other nonlinear decomposition techniques, such as EQS (Extended Quorum System), Disco, and Searchlight (see Figure 1). We measure average discovery latency (slots) and discovered neighbor percentage (percentage of found neighbors) versus duty cycle (percent), node density, and node speed.

**Figure 2:**
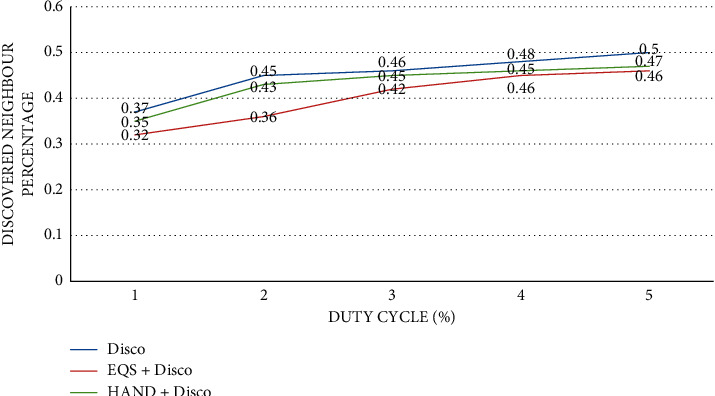
Duty cycle percentage vs. discovered neighbor percentage with pairwise method Disco.

As presented in Figure 2, the impact of duty cycles is observed on discovered neighbor percentage. The duty cycle (%) is provided in the horizontal axis while the vertical axis shows the discovered neighbor [61] percentage. The performance of the proposed HAND protocol along with the Disco pairwise method in terms of discovered neighbor percentage is compared against state-of-the-art methods such as Disco and EQS + Disco methods. An important observation is that duty cycles have an impact on the discovered neighbor percentage. As the duty cycles (%) increase, proportionately the discovered neighbor percentage is increased. The HAND + Disco approach outperforms EQS + Disco method due to its strategy in the usage of discovery schedules efficiently besides maintaining the integrity of wake-up schedules of nodes [62].

**Figure 3:**
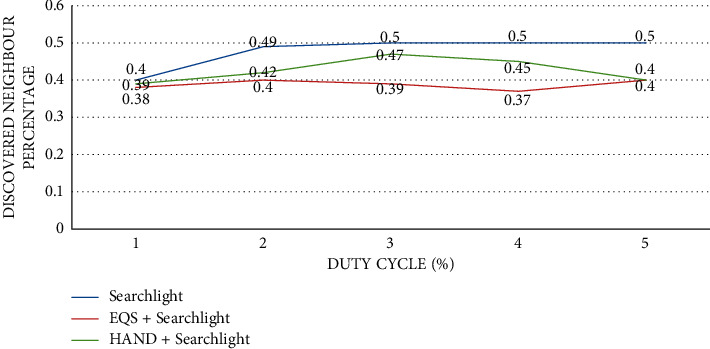
Duty cycle percentage vs. discovered neighbor percentage with pairwise method Searchlight.

As presented in Figure 3, the impact of duty cycles is observed on discovered neighbor percentage with the pairwise method as Searchlight. The duty cycle (%) is provided in the horizontal axis while the vertical axis shows the discovered neighbor percentage. The performance of the proposed HAND protocol along with the Searchlight pairwise method in terms of discovered neighbor percentage [58] is compared against state-of-the-art methods such as Disco and EQS + Disco methods. An important observation is that duty cycles have an impact on the discovered neighbor percentage. As the duty cycles (%) increase, proportionately the discovered neighbor percentage is increased. The HAND + Searchlight approach outperforms the EQS + Searchlight method due to its strategy in the usage of discovery schedules efficiently besides maintaining the integrity of wake-up schedules of nodes.

**Figure 4:**
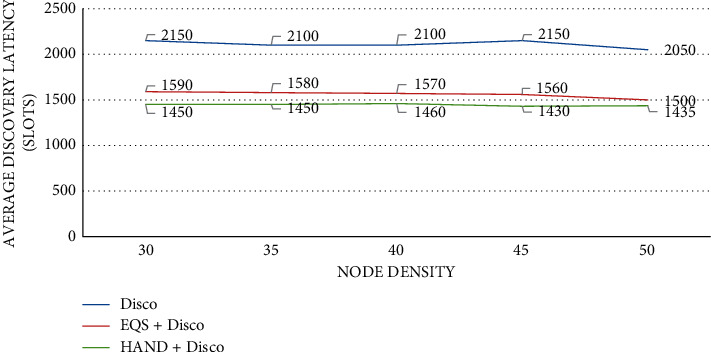
Node density vs. average discovery latency (slots) with Disco as the pairwise method.

As presented in Figure 4, the impact of node density is observed on average discovery latency (slots) with the pairwise method as Disco. The node density is provided in the horizontal axis while the vertical axis shows the average discovery latency (slots). The performance of the proposed HAND protocol along with the Disco pairwise method in terms of average discovery latency (slots) is compared against state-of-the-art methods such as Disco and EQS + Disco methods. An important observation is that node density has an impact on the average discovery latency (slots). As the node density increases, the average discovery latency (slots) is slightly changed. The HAND + Disco approach outperforms the Disco and EQS + Disco methods due to its strategy in the usage of discovery schedules efficiently besides maintaining the integrity of wake-up schedules of nodes.

**Figure 5:**
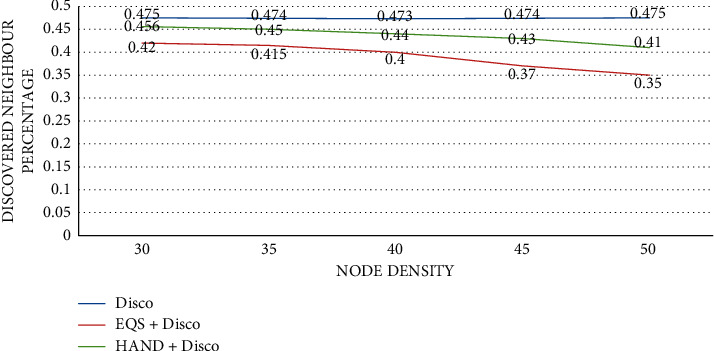
Node density vs. discovered neighbor percentage with Disco as the pairwise method.

As presented in Figure 5, the impact of node density is observed on discovered neighbor percentage with the pairwise method as Disco. The node density is provided in the horizontal axis while the vertical axis shows the discovered neighbor percentage. The performance of the proposed HAND protocol along with the Disco pairwise method in terms of discovered neighbor percentage is compared against the state-of-the-art methods such as Disco and EQS + Disco methods. An important observation is that node density slightly impacts the discovered neighbor percentage. As the node density increases, the discovered neighbor percentage is slightly changed. The HAND + Disco approach outperforms the EQS + Disco method due to its strategy in the usage of discovery schedules efficiently besides maintaining the integrity of wake-up schedules of nodes.

**Figure 6:**
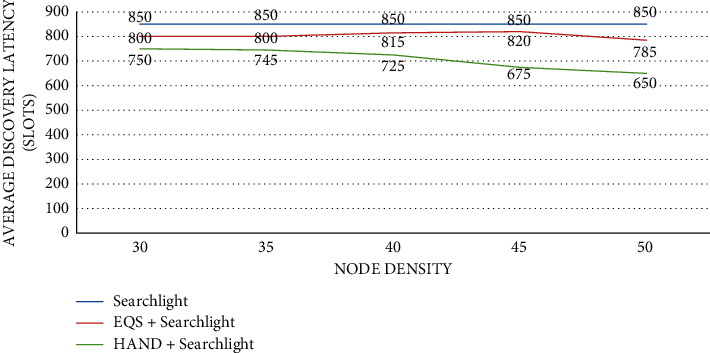
Node density vs. average discovery latency (slots) with Searchlight as the pairwise method.

As presented in Figure 6, the impact of node density is observed on average discovery latency (slots) with the pairwise method as Searchlight. The node density is provided in the horizontal axis while the vertical axis shows the average discovery latency (slots). The performance of the proposed HAND protocol along with the Searchlight pairwise method in terms of average discovery latency (slots) is compared against the state-of-the-art methods such as Searchlight and EQS + Searchlight methods. An important observation is that node density has an impact on the average discovery latency (slots). As the node density increases, the average discovery latency (slots) is significantly changed. The HAND + Searchlight approach outperforms the EQS + Searchlight method due to its strategy in the usage of discovery schedules efficiently besides maintaining the integrity of wake-up schedules of nodes.

**Figure 7:**
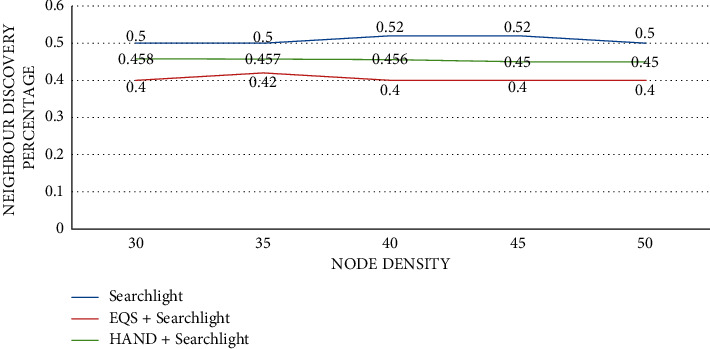
Node density vs. neighbor discovery percentage with Searchlight as the pairwise method.

As presented in Figure 7, the impact of node density is observed on neighbor discovery percentage with the pairwise method as Searchlight. The node density is provided in the horizontal axis while the vertical axis shows the neighbor discovery percentage. The performance of the proposed HAND protocol along with the Disco pairwise method in terms of neighbor discovery percentage is compared against state-of-the-art methods such as Searchlight and EQS + Searchlight methods. A significant observation is that the number of nodes in a cluster influences the average discovery delay (slots). As the number of nodes in a network grows, the percentage of neighbors discovered changes substantially. The HAND + Searchlight approach outperforms EQS + Searchlight method due to its strategy in the usage of discovery schedules efficiently besides maintaining the integrity of wake-up schedules of nodes.

**Figure 8:**
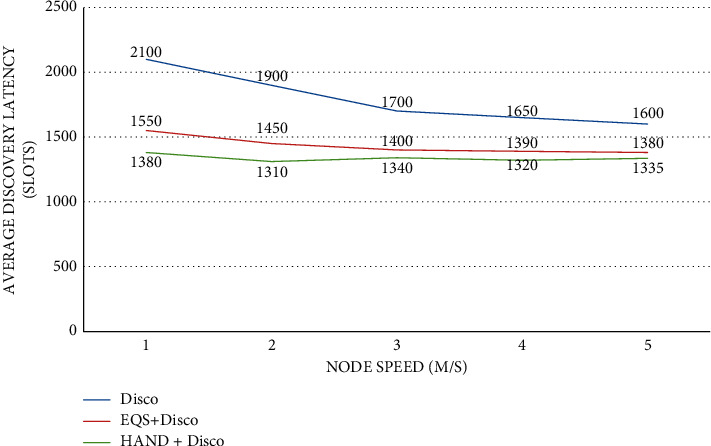
Node speed vs. average discovery latency (slots) with Disco as the pairwise method.

As presented in Figure 8, the impact of node speed is observed on average discovery latency (slots) with the pairwise method as Disco. The node speed is provided in the horizontal axis while the vertical axis shows the average discovery latency (slots). The performance of the proposed HAND protocol along with the Disco pairwise method in terms of average discovery latency (slots) is compared against the state-of-the-art methods such as Disco and EQS + Disco methods. An important observation is that node speed has an impact on the average discovery latency (slots). As the node speed increases, the average discovery latency (slots) is significantly changed. The HAND + Disco approach outperforms Disco and EQS + Disco methods due to its strategy in the usage of discovery schedules efficiently besides maintaining the integrity of wake-up schedules of nodes.

**Figure 9:**
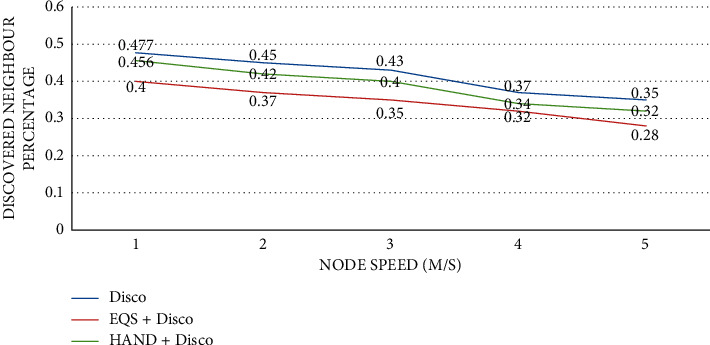
Node speed vs. discovered neighbor percentage with Disco as the pairwise method.

As presented in Figure 9, the impact of node speed is observed on discovered neighbor percentage with the pairwise method as Disco. The horizontal axis indicates the pace of the node, while the vertical axis indicates the proportion of neighbors that have been detected. The performance of the proposed HAND protocol along with the Disco pairwise method in terms of discovered neighbor percentage is compared against the state-of-the-art methods such as Disco and EQS + Disco methods. An important observation is that node speed has an impact on the discovered neighbor percentage. As the node speed increases, the discovered neighbor percentage is significantly changed. The HAND + Disco approach outperforms EQS + Disco method due to its strategy in the usage of discovery schedules efficiently besides maintaining the integrity of wake-up schedules of nodes.

**Figure 10:**
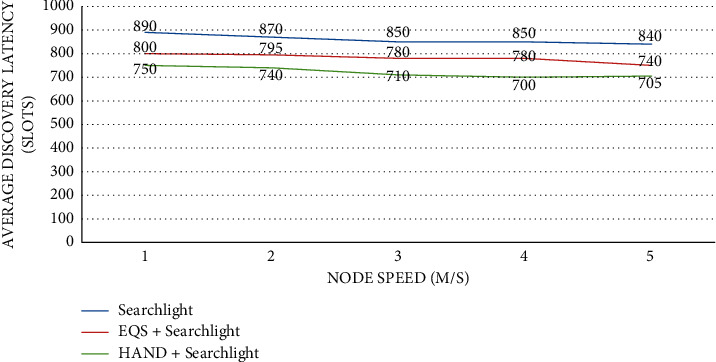
Node speed vs. average discovery latency (slots) with Searchlight as the pairwise method.

As presented in Figure 10, the impact of node speed is observed on average discovery latency (slots) with the pairwise method as Searchlight. The node speed is provided in the horizontal axis while the vertical axis shows the average discovery latency (slots). The performance of the proposed HAND protocol along with the Searchlight pairwise method in terms of average discovery latency (slots) is compared against the state-of-the-art methods such as Searchlight and EQS + Searchlight methods. An important observation is that node speed impacts the average discovery latency (slots). As the node speed increases, the average discovery latency (slots) significantly changes. The HAND + Searchlight approach outperforms Searchlight and EQS + Searchlight methods due to its strategy in the usage of discovery schedules efficiently besides maintaining the integrity of wake-up schedules of nodes.

**Figure 11:**
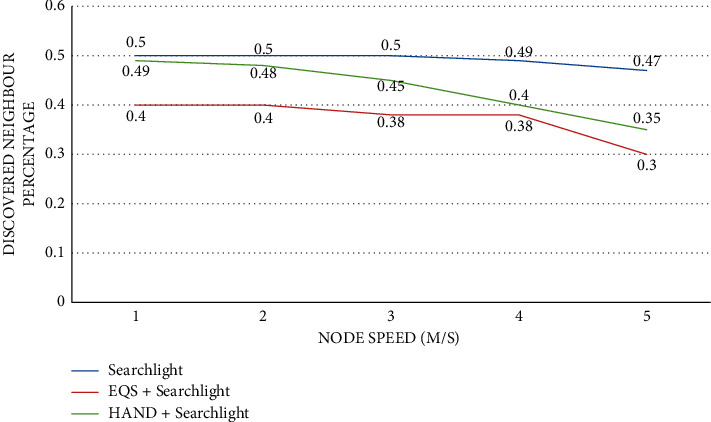
Node speed vs. discovered neighbor percentage with Searchlight as the pairwise method.

As presented in Figure 11, the impact of node speed is observed on discovered neighbor percentage with the pairwise method as Searchlight. The node speed is provided in the horizontal axis while the vertical axis shows the discovered neighbor percentage. The performance of the proposed HAND protocol along with the Searchlight pairwise method in terms of discovered neighbor percentage is compared against the state-of-the-art methods such as Searchlight and EQS + Searchlight methods. An important observation is that node speed has an impact on the discovered neighbor percentage. As the node speed increases, the discovered neighbor percentage is significantly changed. The HAND + Searchlight approach outperforms EQS + Searchlight method due to its strategy in the usage of discovery schedules efficiently besides maintaining the integrity of wake-up schedules of nodes.  Table 1 includes the intrusion and attack implementation.

They are responsible for executing network level and device level intrusion detection at the diagnostic and aggregation levels of the network, respectively, using mobile agents deployed in the network's sensor and cluster-head nodes. The WBAN sensing devices are classified as category A and the other kinds of smart medical devices are classified as category *B*. This is done for the purpose of clarity and to distinguish between them. The following is a description of the detection procedure followed by the mobile agents: training sets for network and device-level detection are preloaded into the cluster heads (CHs) before they are used.

- In the category *A* network (WBANs), two different instances of mobile code instance known as sensor agents (SAs) are formed and trained for network and device-level detection, respectively, in two independent instances of mobile code instance.
- Both instances of SAs are replicated until there is a single network and device detection sensor agent for every group of devices in the cluster, at which point the process is completed. To accomplish localized detection, the SAs are propagated across the WBAN to all trajectories, regardless of their location.
- The SAs only travel with the current state of their trained detection algorithm, not the training dataset, which is not included. This guarantees that agents are kept to a bare minimum in terms of size, hence reducing communication overhead with each hop. The SA aggregates network activity or device data upon arrival to a sensor node, depending on its function as a network intrusion detection agent or as a device intrusion detection agent.
- SA passes aggregated information through its detection algorithm and categorizes them as either benign, in which case it migrates to the next node, malevolent, in which case it triggers an alert, or suspicious, in which case an intervention request is sent to the central hub. In the event that a CH gets a request for intervention, a special agent is created and deployed to sweep the whole cluster for network activity or device data. It is useful when dealing with skilled adversaries that want to disseminate their attack vectors across the network.
- After the SA has sent the aggregated information to the CH, the CH processes the information using an instance of the algorithm that has been trained for cluster-scale detection. As a consequence, the classification result is binary, meaning that it is either benign or malignant. If the WBAN is configured to use just one cluster-head agent (CH), an instance of a static cluster-head agent (CH) is launched to conduct localized network intrusion detection at predetermined intervals.
- Device-level intrusion detection is not performed at this level of the network hierarchy since the cluster heads do not collect any information.

## 5. Conclusion and Future Work

In this paper, a hybrid ND protocol is proposed that combines the feature of two categories of ND to reap benefits of both and get rid of the limitations of both. The current ND approaches that are indirect in nature are categorized into methods of removal of active slots from wake-up schedules and intelligent addition of new slots. On the one hand, the article gives a study of IoT-enabled medical imaging, which includes a thorough survey on the sources of medical imaging, and on the other hand, it presents a review of IoT engagement in medical imaging technology. Furthermore, the study discusses the relevance and limitations of IoT-enabled medical imaging management and monitoring, as well as the countermeasures to be used in the event of a failure. The article predicts that the Internet of Things (IoT) in medical imaging will improve the quality of the service and lower the cost of the services and minimize vexation, fatigue, and the amount of time required for the diagnosis of a condition. As a result, an efficient and effective medical sector provided by IoT would be made possible by correct design, ideal management, compression, and encryption techniques. Further research into the various encryption techniques will be conducted in order to create a safe IoT-enabled medical sector with the least amount of hacking and abuse of medical data.

The two methods are found to have certain drawbacks. The first category disturbs the original integrity of wake-up schedules leading to reduced chances of discovering new nodes in WSN as neighbors. When the second category is followed, it may have inefficient slots in the wake-up schedules leading to performance degradation. Making a hybrid is achieved by introducing virtual nodes that help maximize performance by ensuring the original integrity of wake-up schedules and adding efficient active slots. Thus, a Hybrid Approach to Neighbor Discovery (HAND) protocol is realized in WSN. The simulation study revealed that HAND outperforms the existing indirect ND models. In the future, we intend to improve our protocol by using the information of discovered neighbors and replan ND leading to minimize latency and improve energy efficiency further.

## Figures and Tables

**Algorithm 1:**
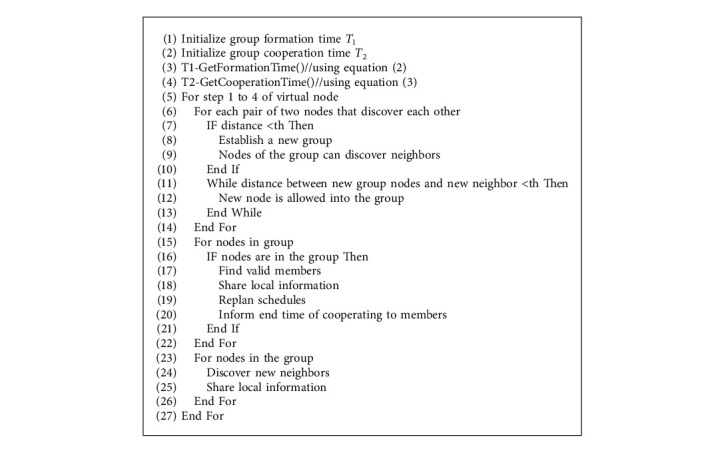
Group formation and group cooperation (GFGC).

**Table 1:** Types of intrusion and the attack implemented in the proposed work.

Attack	Category	Launch point (s)	Implementation
Sender radio exhaustion	DoS	During packet transmission	Increasing the sampling and transmission rate of a sensing device exponentially
Receiver radio exhaustion	DoS	During packet transmission	A group of malicious sensors target a victim and flood it with data packets
Decoy packets	DoS	During packet transmission	Sent noise to the CH as opposed to the meaningful data packet
Sink-holes	DoS	Both packet reception and transmission	Selective or no transmission of sensed data to CH
Data falsification	Data-driven attack	Right after an incoming sample of sensor/image data	Modifying sensed data before transmission to the CH
Illegal transmitter	Privacy breach	During packet transmission	Transmission of data to an illegal destination other than the CH

## Data Availability

The data that support the findings of this study are available on request from the corresponding author.
